# Bridging Housing and Health Inequities: A Qualitative Study on Potential Solutions From the Perspectives of Recently Arrived Migrants and Refugees in Australia

**DOI:** 10.1002/hpja.70225

**Published:** 2026-07-27

**Authors:** Kritika Rana, Jennifer L. Kent, Shameran Slewa‐Younan, Andrew Page

**Affiliations:** ^1^ Translational Health Research Institute, Western Sydney University Campbelltown New South Wales Australia; ^2^ The University of Sydney School of Architecture, Design and Planning, the University of Sydney Sydney New South Wales Australia

**Keywords:** health, housing, migrant, qualitative, refugee, wellbeing

## Abstract

**Issue Addressed:**

Housing is a critical social determinant of health for migrant and refugee populations, with housing inequalities leading to significant health disparities. This study aimed to explore the perspectives of recently arrived migrants and refugees on potential solutions to address housing and related health inequities.

**Methods:**

This qualitative study recruited thirty recently arrived migrants and refugees residing in Greater Western Sydney, Australia, using purposive and snowball sampling primarily through community organisations, settlement and migrant support services, and social media platforms. Participants completed semi‐structured interviews, and an inductive approach to thematic analysis was employed to identify contextual patterns and themes.

**Results:**

Three major themes were identified: (1) Structural and policy reforms to address housing inequities, including simplifying rental processes, regulating rent control and tenant protections, and increasing affordable housing supply; (2) Integrated housing and health support systems for equitable outcomes, encompassing emergency housing support, financial subsidies, health‐focused housing improvements, and integrated housing and mental health services; and (3) Community‐based housing and social integration initiatives for health equity, involving settlement and housing literacy programs, and culturally tailored support and community engagement initiatives.

**Conclusions:**

These findings highlight the importance of multisectoral solutions that combine systemic policy reforms, cross‐sectoral service coordination, and community‐driven initiatives to address the housing and health inequities experienced by recently arrived migrants and refugees.

**So What?:**

Embedding the lived experiences and priorities of these communities within housing and health strategies is essential for advancing health equity and improving outcomes for migrant and refugee populations.

## 1 Introduction

Access to adequate housing is universally acknowledged as a fundamental human right and a critical social determinant of health and wellbeing [1, 2]. The concept of adequate housing extends beyond physical shelter to encompass multiple dimensions, including affordability, security of tenure, accessibility, location, cultural adequacy, habitability, and the availability of services, materials, facilities and infrastructure [3]. These housing attributes significantly influence physical and mental health outcomes, as well as broader aspects of social and economic wellbeing. The social determinants of health framework, which conceptualises health as shaped by the conditions in which people are born, grow, work, live, and age, provides an important lens for understanding these intersecting inequities, as housing constitutes a critical upstream determinant of health outcomes [2].

For migrant and refugee populations, housing plays an essential role during the resettlement process, shaping both immediate health outcomes and influencing long‐term opportunities for integration and social inclusion [4]. However, evidence suggests that migrant and refugee populations are disproportionately affected by the housing crises reportedly impacting high‐income countries, which are marked by declining housing affordability and rising rates of homelessness [5, 6, 7]. Recently arrived migrants and refugees are particularly vulnerable and often face structural barriers to accessing adequate, affordable, and secure housing in the resettlement country, thereby exacerbating health disparities and reinforcing patterns of social and economic disadvantage [5, 8]. Housing insecurity encompasses a spectrum of conditions ranging from housing affordability stress and insecure tenure to overcrowding and homelessness. Definitions of adequate housing may also vary across cultural and policy contexts; for example, practices such as multigenerational living or shared accommodation may be viewed differently across communities. While public health responses to housing insecurity have been increasingly documented in high‐income countries, these approaches have rarely been designed in ways that meaningfully engage migrant and refugee communities or adequately account for the intersecting structural barriers that shape their housing experiences [5, 8].

Australia represents a major resettlement country for migrants and refugees, with more than one‐third of its population born overseas [9]. The ongoing housing crisis has profound implications for the health and wellbeing of marginalised communities, and the population groups that face housing disparities are also found to experience substantial health disparities across housing‐related health outcomes [6, 10]. Existing research has demonstrated how housing inequalities contribute to health disparities among migrant and refugee populations in high‐income countries, including Australia [5, 8]. Our recent mixed‐methods systematic review identified consistent associations between various housing‐related factors, including housing conditions, tenure, insecurity, satisfaction, affordability and neighbourhood conditions, and both physical and mental health outcomes [5]. Among the Australian studies included in the review, three quantitative studies focused specifically on recently arrived humanitarian migrants and demonstrated substantial housing and health inequities affecting migrant and refugee populations [11, 12, 13]. However, none of these studies explored potential solutions to housing‐related health inequities from the perspectives of recently arrived migrants and refugees themselves. These findings are consistent with Australian evidence showing that housing insecurity among culturally and linguistically diverse migrant and refugee communities is not an isolated issue but instead reflects interconnected structural and service‐level factors [14, 15, 16, 17]. These include barriers to navigating housing and support systems, limited access to affordable and appropriate housing, and broader settlement challenges that shape pathways to stability and wellbeing. Especially among some recently arrived migrant and refugee subgroups, these challenges may be compounded by pre‐migration vulnerabilities such as forced displacement and exposure to potentially traumatic events, as well as post‐migration stressors including discrimination and financial hardship, which may contribute to an elevated risk of poor health outcomes in certain subgroups [18, 19]. However, the unique housing and healthcare needs of recently arrived migrants and refugees are often overlooked, as these groups remain underrepresented in both research and policy, leading to a significant gap in understanding and addressing their specific challenges [20].

In line with prior research and the United Nations High Commissioner for Refugees' (UNHCR) definition, “migrants and refugees” in this study refer to groups of people travelling in mixed movements, who may have multiple, overlapping reasons for moving [5, 6, 21]. However, it is important to acknowledge that individual experiences may be shaped by contextual differences in migration pathways, access to settlement support, and prior exposure to adversity. Refugees and humanitarian entrants may have been exposed to forced displacement, persecution, and trauma prior to arrival [18], and may receive some formal settlement support upon resettlement in Australia, including case management, housing assistance, and social support through programs such as the Settlement Engagement and Transition Support (SETS) program and the Humanitarian Settlement Program (HSP) [22]. However, the duration and availability of such support are time‐limited and not uniformly available across all visa categories. In contrast, other migrant subgroups, including those on student, skilled, or family visas, may arrive through diverse migration pathways and may have more limited access to formal settlement services. It is also important to note the heterogeneity within visa categories as many individuals entering through non‐humanitarian visa pathways, such as family reunion or spouse visas, may share countries of origin and similar pre‐migration experiences with refugees. Therefore, post‐migration challenges, including navigating the private rental market, overcoming discrimination, and managing financial insecurity, are often more nuanced and overlapping experiences than visa categories alone would suggest. In this study, recently arrived migrants and refugees are conceptualised as a broader collective group experiencing shared housing challenges during the early resettlement period, particularly when navigating the complexity of the Australian housing system, which has important implications for both physical and mental health outcomes [5].

Recognising housing as a critical social determinant of health, existing research has documented the housing‐related challenges experienced by migrant and refugee populations [5, 8]. However, there is limited evidence on community‐identified strategies to address the intersection of housing and health for recently arrived migrants and refugees in Australia. While the relationship between housing and health inequities is also increasingly recognised, there remains limited research on potential solutions informed by the lived experiences of migrant and refugee populations [5, 8, 10]. Moreover, existing housing and health policies often overlook the intersectional challenges faced by migrants and refugees and rarely meaningfully engage these communities in the design of interventions [23]. Participatory qualitative research provides a valuable approach to addressing this gap by ensuring that proposed solutions are culturally relevant, contextually appropriate, and reflective of lived realities [24]. Furthermore, gaining insight into how recently arrived migrants and refugees perceive the challenges they encounter, and the solutions they believe would improve their housing and health outcomes, is critical for developing equitable and responsive policies and interventions [25].

Greater Western Sydney (GWS), located in New South Wales, Australia, represents a promising site for this study. The region is home to 2.6 million residents, nearly two in five of whom were born overseas [26, 27]. GWS is also a major settlement area for recently arrived migrants and refugees [27]. According to the 2021 Census, 40.9% of its residents were born overseas, of whom 18% had arrived in Australia within five years prior to 2021 [26]. The region is characterised by significant cultural and linguistic diversity, alongside marked socioeconomic disadvantage [26, 28]. Housing challenges are a major concern in GWS, attributed to the growing housing crisis and rising levels of mortgage and rental stress [29]. Despite these factors, there remains a lack of research examining the housing needs of recently arrived migrant and refugee communities in GWS, or their perceptions of potential strategies to address housing and health inequities. Addressing this research gap is paramount to recognising housing as an infrastructure of health equity.

Therefore, this qualitative study seeks to explore the perspectives of recently arrived migrants and refugees in Australia on potential solutions that can address the housing and related health inequities. Drawing on the lived experiences of participants residing in GWS, this study will identify community‐informed recommendations for housing and public health interventions that can be adopted to reduce these inequities. Guided by the social determinants of health framework, the findings will inform collaborative and equity‐focused approaches to housing and health policy in Australia and other high‐income countries [2].

## 2 Methods

### 2.1 Research Design

A qualitative research design was chosen for its open and exploratory nature, which enabled the collection of in‐depth information on participants' perspectives regarding potential solutions to address housing and health inequities [24]. This study is grounded in the constructivism paradigm, which facilitates the co‐construction of knowledge to generate valuable insights into actionable interventions [30]. The qualitative methods and reporting of results adhere to the Consolidated Criteria for Reporting Qualitative Research (COREQ) [31], a 32‐item checklist used to ensure rigour and transparency in qualitative research (Table S1).

### 2.2 Participant Recruitment

Participant recruitment was conducted using a combination of purposive and snowball sampling techniques [24, 32]. Purposive sampling was employed to specifically identify community organisations, settlement service providers, and migrant support agencies operating within GWS that provide a range of services to recently arrived migrant and refugee populations. Organisations were selected based on their relevance to the study population and their established role in providing settlement, social, health, housing, and community support services. The selected organisations, including community forums, multicultural services, settlement agencies, migrant resource centres, health and social service providers, and refugee support organisations, were contacted and provided with a recruitment flyer. Community leaders and organisation staff also assisted in distributing the recruitment materials through their networks and, in some cases, facilitated connections with potential participants. Moreover, invitations to participate in the study were shared by the research team through social media platforms (e.g., Facebook), university communications, and professional networks. Snowball sampling and word‐of‐mouth techniques were also utilised, encouraging participants to refer friends or family members who might be eligible to participate. The recruitment flyer included a QR code leading to a screening form containing the participant information sheet and a screening questionnaire. Interested respondents completed the screening form, which was used to confirm their eligibility for participation. Eligible participants were: women and men aged 18 years or above who arrived in Australia in the past five years as a migrant or refugee; born in a low‐ and middle‐ income country as defined by the World Bank [33]; currently residing in GWS; had experienced and/or were experiencing challenges with housing in Australia (self‐reported via the screening form); and able to participate in interviews conducted in English language. Individuals who required a translator or interpreter to participate in an English‐language interview were not eligible, and this exclusion criterion was applied due to resource constraints as GWS is home to a population drawn from over 170 countries and more than 100 languages represented [34]. All interested participants who met the eligibility criteria on the screening form were contacted via telephone and email by the primary researcher and invited to participate in a one‐hour semi‐structured interview. Written informed consent was obtained from each participant prior to their commencement in the study. Ethics approval for this study was obtained from the Western Sydney University Human Research Ethics Committee (reference number H15923). A $50 gift voucher was provided to each participant to acknowledge the time taken to participate in the interview. Participant recruitment and data collection continued until data saturation was achieved, the point where all relevant dimensions of interest had been explored [35].

### 2.3 Data Collection

In‐depth semi‐structured interviews were conducted between May and September 2024 by a trained qualitative researcher. All participants opted for online interviews conducted via videoconferencing (Zoom), although face‐to‐face interviews were also available. Each interview was scheduled for approximately one hour and was guided by a semi‐structured interview guide developed based on a comprehensive review of the literature. The key domains covered in the interview guide included participants' housing experiences and challenges since arriving in Australia, the impact of housing experiences on physical and mental health and wellbeing, and perspectives on potential solutions to address housing and health inequities among recently arrived migrants and refugees. The semi‐structured interview process allowed participants to respond freely to open‐ended questions, and follow‐up probes were used to explore additional topics and encourage deeper discussion [24]. Reflective memos were documented after each interview, and debriefing sessions were organised with the research team to assess the completeness of data and identify new areas to explore in subsequent interviews. All interviews were audio‐recorded and transcribed verbatim utilising a professional transcription service to ensure accuracy. Member checking was conducted by returning transcripts to participants for review and feedback. Participants confirmed the accuracy of their transcripts, with minor corrections relating to wording and clarification of meaning incorporated into the final transcripts. Additionally, a sociodemographic questionnaire was administered separately through Qualtrics online survey software (Provo, UT, USA) to collect participant background information and describe the sociodemographic characteristics of the study sample. The questionnaire was completed by eligible participants after informed consent was obtained and prior to participation in the interview.

### 2.4 Data Analysis

This qualitative study employed an inductive approach to thematic analysis to identify and analyse contextual patterns and themes [36, 37]. This approach enabled codes and themes to be generated directly from the interview data, allowing patterns and meanings to emerge organically from participants' accounts. The analysis followed the six‐phase process outlined by Braun and Clarke [36]: familiarisation with the data, generating initial codes, searching for themes, reviewing themes, defining and naming themes, and producing the report. Interview transcripts were imported into NVivo qualitative data analysis software (Version 15, QSR International, Cambridge, MA, USA), and all transcripts were read and re‐read to become thoroughly immersed in the data. The initial coding of the data was performed by one researcher (K.R.), who systematically identified and labelled relevant features of the data indicative of emerging themes or patterns. These codes were then independently reviewed by a second researcher (J.L.K.), who assessed their consistency and coverage, and discrepancies were resolved through discussion to ensure credibility. The initial codes were then grouped into meaningful clusters reflecting broader patterns and iteratively reviewed and refined by one researcher (K.R.) to generate coherent themes and subthemes. The themes and subthemes were subsequently finalised through consensus among the researchers. Reflexivity was maintained throughout the analysis through the use of reflective memos, regular team debriefing sessions, and critical discussion of interpretive assumptions and potential researcher bias. Finally, the themes and subthemes were organised into a narrative addressing the research question, supported by participant quotes, with pseudonyms used to ensure confidentiality and anonymity.

### 2.5 Researcher Positionality and Trustworthiness

The research team comprised an interdisciplinary group with expertise in public health, urban studies, and migrant health, as well as extensive experience working with culturally and linguistically diverse populations, including migrants and refugees. Within the team, one researcher (K.R.) has lived experience as an immigrant, and another researcher (S.S‐Y.) is the child of immigrants. These perspectives informed key aspects of the research process, including study design, data collection, and interpretation of participant narratives. Consistent with a constructivist epistemological approach, this study acknowledges that the researchers' positionalities, including their professional backgrounds, personal experiences, values and social positions, inevitably shape the research process and the co‐construction of knowledge with participants [30]. Reflexivity was therefore actively employed throughout all stages of the study to critically examine and account for these influences and to enhance methodological rigour. Trustworthiness was established in accordance with Lincoln and Guba's framework, encompassing credibility, transferability, dependability, and confirmability [38]. Credibility was enhanced through iterative coding, member checking, and peer debriefing, which supported accurate and nuanced representation of participants' experiences and perspectives. Transferability was facilitated through rich contextual descriptions of participant characteristics, recruitment methods, and the study setting, enabling assessment of the potential applicability of the findings to other contexts, including migrants and refugees in other high‐income countries. Dependability was strengthened through comprehensive documentation of the research process, including interview procedures, coding decisions, and research team discussions, thereby providing a transparent and traceable analytic pathway. Confirmability was supported through ongoing reflexive practice, maintenance of an audit trail, and explicit consideration of researcher positionality, ensuring that interpretations remained grounded in participants' accounts rather than researcher assumptions or biases.

## 3 Results

Thirty recently arrived migrants and refugees residing in GWS participated in online interviews, with an average interview duration of 36 min. The majority of participants were aged 18–24 years (36.6%), male (53.4%), single (60%), had a postgraduate level of education (46.6%), and held temporary resident status in Australia (66.7%). The average length of stay in Australia was 1.4 years, and all participants were living in rental accommodation. Detailed participant characteristics are presented in Table 1.

**TABLE 1:** Socio‐demographic characteristics of the study participants (*n* = 30).

Characteristics	*N* (%)
Age group (years)	
18–24	11 (36.6%)
25–29	5 (16.7%)
30–34	8 (26.7%)
35–39	3 (10.0%)
40–49	3 (10.0%)
Gender	
Female	14 (46.6%)
Male	16 (53.4%)
Country of birth	
Iran	5 (16.7%)
Nepal	5 (16.7%)
Bangladesh	5 (16.7%)
Syria	5 (16.7%)
Nigeria	3 (10.0%)
Afghanistan	2 (6.7%)
India	2 (6.7%)
China	1 (3.3%)
Vietnam	1 (3.3%)
Myanmar	1 (3.3%)
Geographical region of birth	
Middle East	12 (40.0%)
South Asia	12 (40.0%)
East Asia	3 (10.0%)
Africa	3 (10.0%)
Marital status	
Married	12 (40.0%)
Single	18 (60.0%)
Education level	
Year 12 or below	5 (16.7%)
Undergraduate	11 (36.6%)
Postgraduate	14 (46.6%)
Residency status in Australia	
Temporary resident	20 (66.7%)
Student visa	18 (60.0%)
Temporary graduate visa	2 (6.7%)
Permanent resident	10 (33.3%)
Refugee visa	4 (13.3%)
Global special humanitarian visa	4 (13.3%)
Skilled independent visa	1 (3.3%)
Australian citizen	1 (3.3%)
Length of stay in Australia	
Less than 1 year	11 (36.7%)
1–2 years	13 (43.3%)
3–5 years	6 (20.0%)

As shown in Table 2, the thematic analysis of the interview data generated three major themes: (1) Structural and policy reforms to address housing inequities; (2) Integrated housing and health support systems for equitable outcomes; (3) Community‐based housing and social integration initiatives for health equity.

**TABLE 2:** Themes and sub‐themes identified from thematic analysis.

Themes	Sub‐themes	Illustrative Quote
Theme 1: Structural and policy reforms to address housing inequities	Subtheme 1: Simplified and flexible rental processes	*“The government can set up a housing authority that can check people inflating prices without house standards” (Seun, male, 28 years old)*
	Subtheme 2: Implementing rent control and tenant protections	
	Subtheme 3: Expanding access to affordable housing supply	
Theme 2: Integrated housing and health support systems for equitable outcomes	Subtheme 1: Transitional and emergency housing support	*“They tell us about, like, if you are not feeling good, there is a mental health service, but they are not looking at the root causes…Help me in housing, I wouldn't need a mental health service.” (Mehreen, female, 28 years old)*
	Subtheme 2: Financial subsidies for housing and health	
	Subtheme 3: Health and wellbeing focused housing interventions	
	Subtheme 4: Integrated housing and mental health services	
Theme 3: Community‐based housing and social integration initiatives for health equity	Subtheme 1: Settlement and housing literacy programs	*“The government or stakeholders can consult community representatives, or incorporate some services, some workshops in the local community centre.” (Quyen, female, 19 years old)*
	Subtheme 2: Culturally tailored support services and community engagement initiatives	

### 3.1 Theme 1: Structural and Policy Reforms to Address Housing Inequities

Participants consistently framed housing inequities as structurally produced rather than attributable to individual shortcomings or personal circumstances. This pattern was analytically significant, as it held across country of origin, visa category, and length of stay in Australia. Participants converged in their accounts of the conditions generating housing insecurity, as well as in their identification of systemic interventions required to address these conditions. Participants' accounts were shaped by shared emotional experiences, including frustration at the perceived arbitrariness of rental requirements, anxiety associated with financial precarity, and a sense of injustice regarding regulatory conditions that appeared to favour landlords over tenants. Participants' calls for policy reform focused on simplifying and rendering more flexible rental application process, implementing rent control and stronger tenant protections, and expanding the supply of affordable housing to improve accessibility and stability for recently arrived migrants and refugees.

#### 3.1.1 Subtheme 1: Simplified and Flexible Rental Processes

Participants emphasised the need for streamlined and adaptable rental processes to address the unique housing challenges faced by recently arrived migrants and refugees. Many participants highlighted the importance of transparent, simplified, and accessible rental procedures to ensure fairness, along with reducing rental application requirements, such as local references or extensive rental histories, to make it easier for new arrivals to secure housing upon arrival in Australia (Quote‐1).

Quote‐1: “Immigrants that come here have problems in housing because they haven't any payslip or rental background. Government can solve these problems with establishing laws to omit some of these things for new immigrants.” (Malik, male, 42 years old)

Another key recommendation was improving access to rental opportunities through a government‐designed housing platform specifically tailored for migrants, which would help newcomers more easily navigate the rental market and find accommodation (Quote‐2). Additionally, participants advocated for government‐backed documentation to validate newcomers' status, which could facilitate rental approvals and improve housing access.

Quote‐2: “There are many applications now, but it's not for newcomer. Residents can use these websites and application, but if the government can design application just for newcomer to find accommodation.” (Mehdi, male, 37 years old)

Participants also suggested implementing flexible payment schedules, such as monthly instead of weekly rent payments, to accommodate financial instability (Quote‐3), as well as reducing bond requirements to lessen the upfront financial burden for new arrivals (Quote‐4).

Quote‐3: “If they're not able to pay rent for the week, allow them to pay it monthly instead of weekly or fortnightly. Give them the flexibility and not stick by the contract.” (Abhishek, male, 24 years old)

Quote‐4: “There should be some consideration for migrants, especially who just arrived. If they minimise the bond to one week, at least for the migrants, that would be great.” (Farnaz, female, 28 years old)

The suggested measures were seen as critical to easing the immediate psychological stress of securing housing, preventing overcrowding or homelessness, and improving associated health outcomes (Quote‐5).

Quote‐5: “If it is easy, we can prevent overcrowding living in a house. If I would have got a rent before they come in here, it is easy to prevent overcrowding. We searched for three months and later found a house.” (Satya, male, 21 years old)

#### 3.1.2 Subtheme 2: Implementing Rent Control and Tenant Protections

Many participants advocated for government interventions to regulate rent prices and protect tenants from exploitative practices. Proposed solutions included capping annual rent increases and establishing guidelines to prevent sudden and excessive rent hikes (Quote‐6).

Quote‐6: “They should put a cap on how much they can increase rent. I've seen house prices go up in three to four months and every time, it goes up $5, $10. At the end of the year, it goes up $50 to $100. They should put a cap on the rental market.” (Nabin, male, 19 years old)

Participants also recommended stricter oversight of rental properties to ensure landlords maintain safe and habitable living conditions, including enforcing standards for housing quality relative to price (Quote‐7).

Quote‐7: “The government can set up a housing authority that can check people inflating prices without house standards. You're putting your price for 300, but equipment is not working, the house is not tidy, it's not up to the standard, you should put that agency to check. If anybody is complaining, you can check with the real estate or owner to reduce the price because it's not up to the standard.” (Seun, male, 28 years old)

Other recommendations included policies to prevent arbitrary rent increases and unfair evictions, ensuring equitable rental agreements, and establishing oversight bodies to address grievances (Quote‐8).

Quote‐8: “Manage the price of renting. The government must have a policy and law and regulation. It is unfair that the agent and the owner have a right to increase if they want. As I know, there is no comprehensive policy about this matter.” (Mehdi, male, 37 years old)

Some participants highlighted the importance of strengthening tenant protections at the end of leases to prevent exploitative practices, such as unjustified evictions or excessive bond deductions. Participants regarded these reforms as essential to mitigating unnecessary emotional and financial stress, promoting housing stability, and supporting mental wellbeing, particularly for recently arrived migrants and refugees (Quote‐9).

Quote‐9: “If they decreased the price range, it would be best. You can get a house through an app or real estate or friends, but if it's not within your price range, it's not possible to stay, especially for students or migrants, $1,400 or $1,500 per month is too much.” (Ahmad, male, 30 years old)

#### 3.1.3 Subtheme 3: Expanding Access to Affordable Housing Supply

Participants consistently identified the severe shortage of affordable housing as a key driver of housing inequities, urging the government to invest in large‐scale affordable housing projects. Suggestions included the construction of prefabricated or modular housing to rapidly expand supply and efficiently utilise available land (Quote‐10).

Quote‐10: “The government should just build more houses. I was researching house plan, China is currently doing, it's called a prefabricated house. If they can implement that in Australia, it will be very good because they have a lot of land.” (Seun, male, 28 years old)

Participants also stressed the importance of prioritising housing allocations for marginalised groups, particularly low‐income families experiencing housing insecurity (Quote‐11).

Quote‐11: “The government have to allocate enough budget for new buildings, new apartments. I think Sydney have many places, and government can allocate this budget for the unprivileged circle of society, the poor people, unprivileged people, students…I have seen a mother with baby crying because they don't have a place. I have seen a father that think that all of the house they see is not affordable.” (Mehdi, male, 37 years old)

Some participants emphasised the need for housing development to keep pace with the growing housing demand in order to prevent exacerbating affordability issues (Quote‐12).

Quote‐12: “They can build more homes because that's the way they can decrease the price range, because every year, they're accepting visas from students or migrants. If you are going to bring them in your country, you have to make a place for them.” (Ahmad, male, 30 years old)

Moreover, expanding the housing supply was seen as a crucial strategy to reduce competition, lower rental costs, and alleviate financial stress, ultimately improving both financial and mental wellbeing (Quote‐13).

Quote‐13: “By increasing the number of houses or apartments, it would be really helpful. Not just immigrants, it would be valuable for Australians too, because it would reduce the price of houses and the renting. If the necessity becomes lower of available accommodation, definitely the price would be decreased.” (Hamid, male, 31 years old)

Other proposed suggestions included offering subsidies or tax incentives to landlords who provide affordable rental housing, investing in public housing programs, and incentivising the construction of affordable housing units. Moreover, participants viewed the increased availability of housing as vital for the health and wellbeing of the broader Australian population as well, helping to stabilise the rental market and promoting fair and equitable access to housing (Quote‐14).

Quote‐14: “If they can build more houses for people to live in, people will rent it. Because I was told that because there are so many migrants and the house available is not really much, that is why the prices are high. But if they can make available more houses, it'll be easier for people to look elsewhere.” (Temi, male, 30 years old)

### 3.2 Theme 2: Integrated Housing and Health Support Systems for Equitable Outcomes

A second overarching pattern was participants' understanding of housing instability and health outcomes as a single cascading system in which inadequate housing directly produces health vulnerability. Rather than conceptualising housing and health as discrete domains requiring separate policy responses, participants articulated an integrated perspective in which housing provision operates as a form of health support. Participants identified multiple pathways through which housing insecurity produces health impacts, including financial trade‐offs that restrict access to healthcare, overcrowded living conditions associated with increased infectious disease and mental health risks, and the ongoing psychological stress of housing precarity. Participants consistently emphasised the critical need for coordinated interventions addressing the intersection of housing instability and health disparities, particularly among recently arrived migrants and refugees. Proposed solutions included emergency housing support, financial subsidies, health‐focused housing improvements, and integrated housing and mental health services to mitigate the compounded stressors of insecure housing and poor health outcomes.

#### 3.2.1 Subtheme 1: Transitional and Emergency Housing Support

Participants highlighted the need for transitional housing support to ease the immediate stress of securing accommodation for recently arrived migrants and refugees. While some participants on certain humanitarian or refugee visa subclasses reported receiving temporary housing upon arrival in Australia, others noted that the support was not consistently available across all visa types, and where provided, it was often limited in duration to meet the needs of new arrivals. Some participants also recommended extending the duration of temporary housing provided by settlement organisations, arguing that the standard one‐month provision was insufficient and advocating for longer transition periods to ensure greater housing stability (Quote‐15).

Quote‐15: “When we first arrived here, the government arranged us to stay at a hotel…and after 28 days we have to look for long‐term accommodation… The time they give is very short. I think at least three should be given.” (Htwe, female, 49 years old)

Many participants receiving settlement support also stressed the importance of providing temporary accommodation for the first few months after arrival, allowing them time to settle and secure long‐term accommodation without undue pressure (Quote‐16).

Quote‐16: “At least for three months, they should support immigrants to settle down and understand their future, and they can manage the future themselves…For the first days, you can manage and settle down, and after that, you will be comfortable to look for a better accommodation for a long‐term situation.” (Hamid, male, 31 years old)

A notable divergence emerged between participants who received formal transitional housing support upon arrival, primarily those on certain humanitarian or refugee visa subclasses, and those who had entered on student, skilled, or family visas and received no such support. While the former group described the inadequacy of the support provided, the latter described the complete absence of a safety net, which generated acute financial and psychological distress during the early settlement period. Across migrant groups without access to formal settlement or housing support, participants emphasised the need for emergency housing programs to assist those at risk of eviction or homelessness and to mitigate the associated mental and physical health impacts (Quote‐17). Fear of eviction was a particularly salient and recurring emotional experience, described as a sustained source of psychological distress that shaped participants' sense of safety and belonging in Australia.

Quote‐17: “We are stressed, what will happen, you get an eviction notice, people are worried. One big fear of migrants is getting an eviction notice because you don't know where to go after that. You don't have anywhere to go.” (Abhishek, male, 24 years old)

#### 3.2.2 Subtheme 2: Financial Subsidies for Housing and Health

Participants emphasised the significant financial barriers to securing stable housing and accessing essential healthcare and advocated for targeted subsidies to alleviate these burdens. Proposed solutions included emergency financial assistance to help cover rental and utility expenses, particularly during the settlement period, as well as government‐backed bond loans (Quote‐18) accessible to all recently arrived migrants and refugees, regardless of visa status.

Quote‐18: “They don't have any program for finding house, but they helped us with bonds. They lent us some money for a bond. I paid the bond back to government, but it was so helpful because without bonds, we cannot rent our house.” (Saba, female, 34 years old)

Many participants described making difficult trade‐offs to afford housing, such as skipping meals or forgoing healthcare, which they recommended should be addressed (Quote‐19 and Quote‐20). This “tip of the ice” metaphor reflects participants' perception that visible housing costs are only the surface‐level problem, while hidden downstream consequences, such as food insecurity, healthcare avoidance, and cumulative financial strain, remain largely unaddressed within policy responses (Quote‐19). The inability to afford dental or dermatological care reflects how housing‐driven financial precarity forecloses access to preventive and primary care, generating a cycle of unmet health needs and compounding psychological stress (Quote‐20). Therefore, participants identified additional support, including financial assistance for health‐related expenses (Quote‐20) and discounts on groceries and transport (Quote‐21), as essential for reducing immediate financial stress and improving access to adequate housing and healthcare.

Quote‐19: “So, the problem mostly is tip of the ice. Housing is expensive, but below the surface of the water, there are so many layers that are compromised because of that tip of the ice.” (Mehreen, female, 28 years old)

Quote‐20: “So, it's also mental stress…If we have skin issues, we need a dermatologist, we cannot go because it's very expensive. If we face any dental issues, we cannot go, like it's very expensive here.” (Tabu, female, 21 years old)

Quote‐21: “But I think also in terms of groceries…there should be some kind of student card. That would be a good idea for migrants or students. It's kind of like a student debit card they can use and get some discounts.” (Abhishek, male, 24 years old)

#### 3.2.3 Subtheme 3: Health and Wellbeing Focused Housing Interventions

Participants highlighted the adverse health impacts of poor housing conditions, such as mould, overcrowding, and inadequate maintenance, and called for housing interventions that prioritise health and wellbeing. Many participants advocated for stronger regulatory oversight through regular government inspections to monitor and address issues such as overcrowding and substandard living conditions, along with enforcing liveability standards and ensuring rental properties meet basic health and safety requirements (Quote‐22).

Quote‐22: “Well, the government should send inspector or government‐related people to check houses, like how many people are residing…I'm living with eight different people. That's not good. So, the government should do something about it.” (Sandeep, male, 20 years old)

Participants also described uninhabitable rentals that jeopardised wellbeing, stressing the need for stricter enforcement of housing regulations and penalties for landlords who fail to meet standards (Quote‐23).

Quote‐23: “They're doing whatever they want. They don't have any control over that. So, I think government should prioritise that you put laws and also implement quickly.” (Fatima, female, 35 years old)

Calls for improved monitoring included enforcing maintenance regulations requiring landlords to make properties safe, habitable, and well‐maintained before renting them out (Quote‐24), as well as increasing public awareness of existing tenant support services, such as reporting mechanisms for housing‐related issues (Quote‐25).

Quote‐24: “I was searching for an apartment, and an apartment, very old, very dirty, it was around 600, and I was like, ‘Are you serious? At least clean it.’ If you put dog in this space, it will never live, never survive. It's crazy… What the government can do is, monitor or control this. Before you put your apartment to rent, fix it at least, make liveable.” (Abbas, male, 26 years old)

Quote‐25: “There's a website called New South Wales Fair Trading, where people can call and report any moulding or the shower was broken, but then the landlord didn't want to fix it, he wants me to pay for it. I think increased accessibility. So, raise awareness that people can call this when there's some conflict, some problems arise” (Quyen, female, 19 years old)

#### 3.2.4 Subtheme 4: Integrated Housing and Mental Health Services

The intersection of housing instability and mental health emerged as a significant concern, with participants advocating for better integration of housing and mental health services. Many participants emphasised the importance of building the capacity of frontline workers to recognise distress and offer appropriate referrals, alongside increasing awareness of existing helplines, such as Lifeline, and ensuring these services are equipped to offer legal advice or direct individuals to support services when housing issues overlap with mental health challenges (Quote‐26).

Quote‐26: “Raise awareness that people can call this when there's some conflict, or some problems arise, and also mental health helpline…There should be someone qualified to give legal advice or direct people to support services or legal services… Specifically when they feel unsafe, give them a place to talk and seek help.” (Quyen, female, 19 years old)

Some participants stressed the importance of addressing the root causes of mental distress, such as housing insecurity, rather than relying solely on reactive mental health interventions (Quote‐27).

Quote‐27: “They tell us about, like, if you are not feeling good, there is a mental health service, but they are not looking at the root causes…There's a big problem here. They're rooted in housing. Help me in housing, I wouldn't need a mental health service.” (Mehreen, female, 28 years old)

Improved guidance and knowledge‐sharing about appropriate support services, including one‐on‐one counselling and clear referral pathways, were identified as critical not only for recently arrived migrants and refugees but also for community members and service providers who support them, in order to better understand available options and navigate both housing and mental health challenges (Quote‐26 and Quote‐28).

Quote‐28: “So, if there is something from the government side, and they can guide us…like one‐to‐one counselling, and then give us the solution. Because now, we don't know any exact institution, if they are offering us something like that. When we came here, we went to a place, and they made us very frustrated…We didn't know that time where to go.” (Farnaz, female, 28 years old)

### 3.3 Theme 3: Community‐Based Housing and Social Integration Initiatives for Health Equity

The third theme highlights the role of cultural, linguistic, and social resources in supporting recently arrived migrants and refugees to effectively navigate Australia's housing system. Participants described how inaccessible information, unfamiliar bureaucratic processes, and culturally mismatched services compounded the material challenges of housing insecurity, generating experiences of confusion, isolation, and anxiety that were distinct from but interwoven with financial hardship. Participants did not frame these barriers as personal deficits requiring individual remediation but instead articulated a structural critique of the assumptions embedded in existing systems and of the failure of those systems to adapt to the realities of cultural and linguistic diversity. Participants consistently highlighted the potential role of community‐driven housing and social integration strategies in addressing health inequities, and advocated for settlement and housing literacy programs, as well as culturally tailored support and community engagement initiatives, to foster equitable outcomes.

#### 3.3.1 Subtheme 1: Settlement and Housing Literacy Programs

Participants emphasised the critical need for comprehensive settlement programs that address both housing literacy and broader integration challenges. Many participants highlighted the value of housing literacy programs as comprehensive educational initiatives designed to improve understanding of the local housing market, rental processes, tenant rights, and available support resources, while simultaneously fostering social connections and peer networks among newcomers to promote not only housing stability but also the overall wellbeing and successful integration (Quote‐29).

Quote‐29: “By educating people and giving them housing literacy…But to make it available to everyone, maybe these services for immigrants can be useful… And maybe people who join these classes might be connected and they can share houses.” (Mehreen, female, 28 years old)

Moreover, some participants recommended the development of community‐driven housing literacy programs, which are locally tailored and culturally sensitive, alongside personalised housing‐focused counselling services to address individual needs and simplify the complex process of finding suitable housing (Quote‐30).

Quote‐30: “It'd be helpful if they let us know from where we can get the house easily. Every person's situation is different. So, if government can counsel us, know my situation, and consult me according to my needs, I think that will be a good solution. Because now we know after spending seven months, what is the actual procedure.” (Farnaz, female, 28 years old)

Participants particularly stressed the importance of mandatory settlement orientation programs covering practical aspects of daily life, including transportation and shopping, to facilitate smoother integration and promote wellbeing (Quote‐31). The need for these programs to be universally accessible was also emphasised, with participants noting that housing literacy should be treated as an essential component of successful integration (Quote‐29 and Quote‐30).

Quote‐31: “The officer said we have some training that the immigrant must do. We need to let them open some other trainings for the immigrants. When they go out, their next problem is traffic, they make the problem for shopping. Each stage they take forward, they make problems. So, make the settlement training for them must – to avoid making those problems.” (Mansur, male, 40 years old)

#### 3.3.2 Subtheme 2: Culturally Tailored Support Services and Community Engagement Initiatives

Participants highlighted the complexities of delivering support to culturally diverse groups and underscored the importance of culturally tailored support services (Quote‐32).

Quote‐32: “Different migrants, they come from different cultures, that's also difficult to manage because if you put all the migrants from different cultures in one place to give some support, there would be more conflict than giving the support.” (Fatima, female, 35 years old)

Many participants advocated for community‐led initiatives developed in consultation with cultural representatives, emphasising the need for linguistically accessible services delivered through trusted local community centres to provide culturally sensitive support and foster social connections (Quote‐33).

Quote‐33: “The government or stakeholders can consult community representatives, or incorporate some services, some workshops in the local community centre. So, migrants can easily access in their languages, talk with people who may have lived experience with housing difficulties. When we deliver these services, it needs to be culturally sensitive…and genuinely empower them maintain their health and wellbeing.” (Quyen, female, 19 years old)

Participants also stressed that such culturally grounded approaches should prioritise empowerment, wellbeing and social inclusion. The transition period upon arrival to Australia was identified as particularly challenging, with language barriers and culture shock exacerbating housing‐related stress and anxiety. Peer‐support volunteer and mentorship programs were widely endorsed, where individuals with shared cultural backgrounds or recent migration experiences could assist newcomers in navigating the housing market (Quote‐34). Moreover, participants recommended community engagement initiatives and cultural and social integration programs to facilitate interactions and connections between recently arrived migrants and refugees and local communities, ultimately promoting social cohesion and overall wellbeing.

Quote‐34: “For newcomers who has a language barrier or culture shock, all need time to adjust to the new environment, to be immersed with the new culture. It's going to be a bit of challenge, stressful moments and anxiety…There's supposed to be some volunteers program that they let locals or people who went through this experience to help others who has the same background or language to help them or navigate how to get a house.” (Marwan, male, 32 years old)

## 4 Discussion

This qualitative study explored the perspectives of recently arrived migrants and refugees on potential solutions that can address the housing and related health inequities in Australia. Three major themes were identified in this study. First, participants emphasised the need for structural and policy reforms to address the housing inequities faced by recently arrived migrants and refugees. Second, the findings highlighted the importance of integrated housing and health support systems to achieve equitable housing and health outcomes. Third, participants underscored the value of community‐based housing and social integration initiatives to foster equitable health outcomes. The analytical contribution of this study extends beyond documenting participants' perspectives on housing needs by illuminating the interpretive frameworks through which recently arrived migrants and refugees understand housing insecurity as a structural problem, make meaning of its health consequences, and articulate the types of systemic change they consider necessary. The three identified pathways further point to the need for coordinated and multisectoral responses that are grounded in the social determinants of health framework and are informed by the lived experiences of migrant and refugee communities [10, 39].

This study contributes to the growing body of Australian evidence by exploring community‐identified solutions to housing‐related health inequities among recently arrived migrants and refugees residing in GWS. Building on existing studies in other Australian contexts [14, 15, 16, 17], this study extends the understanding of how recently arrived migrants and refugees understand and experience the structural determinants of housing‐related health inequities, as well as the structural and policy reforms they view as necessary to address these challenges. While prior research has documented the associations between housing inequalities and adverse health outcomes among migrant and refugee populations, this study sheds light on community‐identified priorities for change [5, 8]. Participants' calls for simplifying rental application procedures, regulating rent control, strengthening tenant protections, and expanding access to affordable housing supply align with existing evidence on the systemic drivers of housing precarity, a condition characterised by housing insecurity, unaffordability and instability [40, 41, 42]. Moreover, the participant‐informed recommendations highlight the need to move beyond individualised or behavioural explanations of housing precarity, advocating instead for structural and policy reforms that address the broader economic and regulatory conditions shaping housing access. Consistent with the Ottawa Charter for Health Promotion action area of building healthy public policy [43], the findings underscore that health promotion must engage directly with the structural conditions, including housing policy, anti‐discrimination legislation, and settlement service systems, that shape the everyday lives of recently arrived migrants and refugees. For policymakers, the study findings underscore the importance of designing housing strategies that account for the compounded barriers experienced by recently arrived migrants and refugees and embedding equity‐focused objectives within housing legislation and planning frameworks [44].

Applying the socioecological model as an organising framework [45], the study findings can be understood across three interconnected levels. At the micro level, participants' accounts reveal how individual and household‐level housing experiences, including overcrowding, unaffordable rent, substandard conditions, and the fear of eviction, directly undermine physical and mental health and generate sustained psychological distress. The “tip of the ice” metaphor described by one participant (Quote‐19) further illustrates that visible housing costs represent only the surface‐level burden, while hidden downstream consequences, including foregone healthcare, food insecurity, and cumulative financial strain, remain largely invisible within existing policy responses. At the meso level, the findings highlight gaps in the coordination and cultural responsiveness of housing, health, and settlement services, underscoring the need for more integrated models of support. This aligns with Australian evidence highlighting the role of service providers as connectors, supporters, and advocates in refugee housing and settlement, and the importance of settlement services literacy in enabling recently arrived migrants and refugees to effectively access and navigate available supports [46, 47]. At the macro level, participants identified the need for structural and policy reforms that address the broader regulatory and economic drivers of housing inequity. Within Australia's housing and settlement policy context, initiatives such as the National Housing Accord and the Housing Australia Future Fund, together with settlement programs including the Humanitarian Settlement Program (HSP) and the Settlement Engagement and Transition Support (SETS) program, provide important components of the current support system for housing and settlement [22, 48]. However, barriers related to housing affordability, service eligibility, and time‐limited settlement assistance continue to contribute to housing‐related health inequities among recently arrived migrants and refugees.

The study findings highlight that efforts to improve housing and health outcomes for recently arrived migrants and refugees must be accompanied by integrated housing and health support systems that concurrently address housing insecurity and health disparities. Participants described the need for coordinated responses that include emergency housing support, targeted financial assistance, housing interventions that support health and wellbeing, and stronger integration of housing and mental health services. Rather than viewing housing and health as discrete domains requiring separate policy responses, participants emphasised their interdependence, with one participant noting that resolving housing insecurity is itself a form of mental health intervention (Quote‐27). This grounding in participant experience provides strong empirical support for the argument that fragmented service delivery in which housing and mental health are addressed through separate systems fails to address the root causes of distress experienced by recently arrived migrants and refugees. These findings also align with growing evidence that fragmented service delivery can exacerbate the vulnerabilities faced by marginalised populations and may undermine the effectiveness of interventions targeting the social determinants of health [49]. Multisectoral collaboration between housing providers, healthcare services, and settlement agencies is therefore essential to addressing these intersecting challenges. Within the Australian context, this could include opportunities to strengthen coordination between public and community housing providers, Local Health District (LHD) refugee health services, and settlement agencies such as Settlement Services International (SSI), to support more integrated and culturally responsive housing, health, and settlement pathways. Embedding trauma‐informed and culturally responsive care within integrated service models is particularly important, given the pre‐migration trauma and multiple resettlement challenges faced by recently arrived migrants and refugees [18]. Furthermore, investing in coordinated support systems can also improve access to essential services and reduce long‐term reliance on crisis systems by directly addressing the structural factors that contribute to poor housing and health outcomes [50].

This study provides valuable insights into the essential role of community‐based housing and social integration initiatives in promoting housing stability, social inclusion and overall wellbeing among recently arrived migrants and refugees. Participants highlighted the importance of accessible settlement and housing literacy programs, culturally tailored support services, peer‐support volunteer and mentorship programs, and opportunities for meaningful community engagement as key pathways for navigating the complex housing system. These findings suggest that policy responses should extend beyond structural reforms to incorporate place‐based, community‐led strategies that build capacity, agency, and resilience within migrant and refugee populations [51]. In line with the Ottawa Charter for Health Promotion action areas of strengthening community action and developing personal skills [43], these approaches position communities as active agents in health promotion rather than passive recipients of services. Translating these recommendations into practice may involve co‐design processes involving recently arrived migrants and refugees in the development of housing literacy programs and culturally tailored settlement supports, lived experience advisory mechanisms embedded within housing and settlement organisations to inform ongoing policy and service design, and participatory action research approaches that engage community members as co‐researchers. These implementation strategies are supported by emerging Australian evidence and align with core health promotion principles of empowerment, participation, and equity [43, 52, 53]. Moreover, strengthening partnerships with multicultural organisations and community leaders to co‐design and deliver linguistically accessible and culturally appropriate settlement supports may help enhance the reach and effectiveness of services for recently arrived communities [53]. By prioritising the lived experiences and strengths of migrant and refugee communities, these community‐based initiatives have the potential to facilitate immediate access to stable housing, as well as foster long‐term integration, social cohesion, and health equity.

This study has some limitations. The sample of 30 recently arrived migrants and refugees was drawn from GWS, a single metropolitan region in Australia. Future research could explore the experiences and perspectives of migrants and refugees in other geographic or policy contexts, particularly in regional and remote areas where housing access and support services may differ. The eligibility criterion requiring participants to take part in English‐language interviews was applied due to resource constraints as more than 100 languages are represented across GWS [34], which may have excluded the perspectives of non‐English speaking individuals who often face even greater systemic barriers to accessing housing [25]. It is acknowledged that language proficiency is itself a key determinant of settlement and housing outcomes, and that those excluded may include recently arrived migrants and refugees with lower English proficiency who are likely to be more reliant on settlement supports and may experience more constrained housing pathways [54]. Future qualitative studies could therefore prioritise multilingual approaches to better capture the diversity of housing experiences among migrant and refugee populations. Moreover, the proposed solutions identified by participants reflect perceived strategies rather than tested interventions to address the housing and related health inequities, and further research is needed to evaluate the feasibility and effectiveness of these approaches in practice.

Despite these limitations, this study reinforces the central role of housing in shaping health outcomes for migrants and refugees and provides actionable insights for addressing the structural drivers of health inequities [5, 8]. By foregrounding lived experience and highlighting community‐informed solutions, the study findings contribute to the evidence base for developing more equitable and inclusive housing and health systems in Australia and other high‐income countries. The perspectives of participants in this study underscore the need for multisectoral solutions that combine systemic policy reforms, cross‐sectoral service coordination, and community‐driven initiatives to address the housing and health inequities experienced by recently arrived migrants and refugees. Embedding the lived experiences and priorities of these communities within housing and health strategies is essential for advancing health equity and improving outcomes for migrant and refugee populations.

## Author Contributions

All authors conceived and designed the study. K.R. was responsible for data collection and data management. The formal data analysis and interpretation of data was led by K.R., supported by J.L.K., S.S.‐Y. and A.P. by providing supervision and validation. K.R. was responsible for project administration and A.P. supported the provision of resources. The manuscript was drafted by K.R. All authors made critical revisions, read, and approved the final manuscript.

## Funding

This study received no external funding. Open access funding was enabled and organised by the Council of Australian University Librarians (CAUL) and its member institutions.

## Ethics Statement

Ethics approval for this study was obtained from the Western Sydney University Human Research Ethics Committee (reference number H15923).

## Consent

All participants included in this study provided written informed consent.

## Conflicts of Interest

The authors declare no conflicts of interest.

## Supporting information


**Table S1:** Consolidated criteria for reporting qualitative research (COREQ) 32‐item checklist.

## Data Availability

The data that support the findings of this study are available on request from the corresponding author. The data are not publicly available as they contain information that could compromise the privacy or consent of study participants.

## References

[1] OHCHR , “Article 11.1 of the International covenant on economic, social and cultural rights,” 2018.

[2] G. Dahlgren and M. Whitehead , European Strategies for Tackling Social Inequities in Health: Levelling Up Part 2 (WHO Regional Office for Europe, 2006).

[3] United Nations , “Annual Progress Report 2014: Implementation of the Strategic Plan (2014–2019), New York,” 2014.

[4] A. Ziersch , E. Miller , M. Baak , and L. Mwanri , “Integration and Social Determinants of Health and Wellbeing for People From Refugee Backgrounds Resettled in a Rural Town in South Australia: A Qualitative Study,” BMC Public Health 20, no. 1 (2020): 1700. 33187489 10.1186/s12889-020-09724-z PMC7663864

[5] K. Rana , J. L. Kent , and A. Page , “Housing Inequalities and Health Outcomes Among Migrant and Refugee Populations in High‐Income Countries: A Mixed‐Methods Systematic Review,” BMC Public Health 25, no. 1 (2025): 1098. 40121396 10.1186/s12889-025-22186-5 PMC11929249

[6] K. Rana , A. Page , J. L. Kent , and A. Arora , “Pathways Linking Housing Inequalities and Health Outcomes Among Migrant and Refugee Populations in High‐Income Countries: A Protocol for a Mixed‐Methods Systematic Review,” International Journal of Environmental Research and Public Health 19, no. 24 (2022): 16627. 36554503 10.3390/ijerph192416627 PMC9779591

[7] W. Cox , “2024 Demographia International Housing Affordability,” 2024, http://demographia.com/dhi.pdf.

[8] A. Ziersch and C. Due , “A Mixed Methods Systematic Review of Studies Examining the Relationship Between Housing and Health for People From Refugee and Asylum Seeking Backgrounds,” Social Science & Medicine 213 (2018): 199–219. 30142501 10.1016/j.socscimed.2018.07.045

[9] Australian Bureau of Statistics , “Migration, Australia,” 2021, https://www.abs.gov.au/statistics/people/population/migration‐australia/latest‐release.

[10] C. B. Swope and D. Hernández , “Housing as a Determinant of Health Equity: A Conceptual Model,” Social Science & Medicine 243 (2019): 112571. 31675514 10.1016/j.socscimed.2019.112571 PMC7146083

[11] D. W. Handiso , J. A. Boyle , E. Paul , F. Shawyer , and J. C. Enticott , “Gender Disparity and Post‐Traumatic Stress Disorder and Elevated Psychological Distress in Humanitarian Migrants Resettled in Australia: The Moderating Role of Socioeconomic Factors,” Epidemiology and Psychiatric Sciences 33 (2024): e60. 39506621 10.1017/S2045796024000489 PMC11561526

[12] D. W. Handiso , E. Paul , J. A. Boyle , F. Shawyer , G. Meadows , and J. C. Enticott , “Trends and Determinants of Mental Illness in Humanitarian Migrants Resettled in Australia: Analysis of Longitudinal Data,” International Journal of Mental Health Nursing 33, no. 5 (2024): 1418–1434. 38651241 10.1111/inm.13327

[13] E. Martino , Y. Li , J. Kali‐Opio , and R. Bentley , “Between Liminality and a New Life in Australia: What Is the Effect of Precarious Housing on the Mental Health of Humanitarian Migrants?,” Cities 131 (2022): 103900.

[14] K. Blackford , G. Crawford , K. McCausland , and Y. Zhao , “Describing Homelessness Risk Among People From Culturally and Linguistically Diverse Backgrounds in Western Australia: A Cluster Analysis Approach,” Health Promotion Journal of Australia 34, no. 4 (2023): 953–962. 36764671 10.1002/hpja.704

[15] E. Connor , K. Blackford , K. McCausland , R. Lobo , and G. Crawford , “Searching for Choice and Control: Western Australian Service Provider Experiences of Health, Housing and Migration,” Health Promotion International 39, no. 3 (2024): daae066, https://pubmed.ncbi.nlm.nih.gov/38902981/. 38902981 10.1093/heapro/daae066

[16] G. Crawford , E. Connor , K. McCausland , K. Reeves , and K. Blackford , “Public Health Interventions to Address Housing and Mental Health Amongst Migrants From Culturally and Linguistically Diverse Backgrounds Living in High‐Income Countries: A Scoping Review,” International Journal of Environmental Research and Public Health 19, no. 24 (2022): 16946. 36554827 10.3390/ijerph192416946 PMC9778908

[17] H. B. Eshetu , K. Blackford , R. Lobo , M. B. Alemu , G. A. Tessema , and G. Crawford , “Housing Insecurity and Health Outcomes Among Migrants From Culturally and Linguistically Diverse Backgrounds in High‐Income Countries: A Scoping Review,” Journal of Immigrant and Minority Health (2026), 10.1007/s10903-026-01849-4. 41569347

[18] W. Chen , B. J. Hall , L. Ling , and A. M. N. Renzaho , “Pre‐Migration and Post‐Migration Factors Associated With Mental Health in Humanitarian Migrants in Australia and the Moderation Effect of Post‐Migration Stressors: Findings From the First Wave Data of the BNLA Cohort Study,” Lancet Psychiatry 4, no. 3 (2017): 218–229. 28161455 10.1016/S2215-0366(17)30032-9

[19] K. Rana , S. Sperandei , S. Munasinghe , J. L. Kent , and A. Page , “Economic Deprivation as a Mediator in the Relationship Between Housing Affordability Stress and Mental and General Health Among Humanitarian Migrants in Australia,” Journal of Epidemiology and Community Health 80, no. 5 (2026): 343–351. 41494841 10.1136/jech-2025-224802

[20] A. Renzaho , M. Polonsky , D. Mellor , and S. Cyril , “Addressing Migration‐Related Social and Health Inequalities in Australia: Call for Research Funding Priorities to Recognise the Needs of Migrant Populations,” Australian Health Review 40, no. 1 (2016): 3–10. 26164042 10.1071/AH14132

[21] United Nations General Assembly , “New York Declaration for Refugees and Migrants: resolution adopted by the General Assembly,” 2016.

[22] J. Abood , M. Polonsky , K. Woodward , Z. Tadjoeddin , and A. M. N. Renzaho , “Settlement Service Provision and Social Capital Among Humanitarian Migrants in Australia: A Service Provider Perspective,” International Journal of Sociology and Social Policy 46, no. 13 (2026): 20–35.

[23] M. Hynie , “The Social Determinants of Refugee Mental Health in the Post‐Migration Context: A Critical Review,” Canadian Journal of Psychiatry. Revue Canadienne de Psychiatrie 63, no. 5 (2017): 297–303. 29202665 10.1177/0706743717746666 PMC5912301

[24] K. Rana , P. Poudel , and R. Chimoriya , “Qualitative Methodology in Translational Health Research: Current Practices and Future Directions,” Healthcare (Basel) 11, no. 19 (2023): 2665. 37830701 10.3390/healthcare11192665 PMC10572630

[25] A. Ziersch , M. Walsh , C. Due , and E. Duivesteyn , “Exploring the Relationship Between Housing and Health for Refugees and Asylum Seekers in South Australia: A Qualitative Study,” International Journal of Environmental Research and Public Health 14, no. 9 (2017): 1036. 28885594 10.3390/ijerph14091036 PMC5615573

[26] Australian Bureau of Statistics , “Greater Western Sydney Region‐ Ancestry. Australian Bureau of Statistics,” 2025, https://profile.id.com.au/cws/language?WebID=10%5C.

[27] Western Sydney University , “About Our Region ‐ Greater Western Sydney 2025.”.

[28] Australian Bureau of Statistics , “Census of Population and Housing: Socio‐Economic Indexes for Areas (SEIFA), Australia, 2016. Canberra: Australian Bureau of Statistics,” 2018, https://www.ausstats.abs.gov.au/Ausstats/subscriber.nsf/0/756EE3DBEFA869EFCA258259000BA746/$File/SEIFA%202016%20Technical%20Paper.pdf.

[29] T. Nance , “Home Truths: The Real Housing Story in Western Sydney.: Centre for Western Sydney, Western Sydney University,” 2023.

[30] M. Burns , J. Bally , M. Burles , L. Holtslander , and S. Peacock , “Constructivist Grounded Theory or Interpretive Phenomenology? Methodological Choices Within Specific Study Contexts,” International Journal of Qualitative Methods 21 (2022): 16094069221077758.

[31] A. Tong , P. Sainsbury , and J. Craig , “Consolidated Criteria for Reporting Qualitative Research (COREQ): A 32‐Item Checklist for Interviews and Focus Groups,” International Journal for Quality in Health Care 19, no. 6 (2007): 349–357. 17872937 10.1093/intqhc/mzm042

[32] P. Liamputtong , Qualitative Research Methods, 4th ed. (Oxford University Press, 2013).

[33] The World Bank , “World Bank Country and Lending Groups. Washington (DC): The World Bank,” 2022.

[34] N. Baroy , “Greater Western Sydney: A First Look at the Data. WESTIR Limited,” 2022.

[35] M. Q. Patton , Qualitative Research and Evaluation Methods, 4th ed. (Sage Publications, 2015).

[36] V. Braun and V. Clarke , “Using Thematic Analysis in Psychology,” Qualitative Research in Psychology 3, no. 2 (2006): 77–101.

[37] P. Liamputtong , Qualitative Research Methods, 5th ed. (Oxford University Press, 2020).

[38] Y. S. Lincoln and E. G. Guba , “But Is It Rigorous? Trustworthiness and Authenticity in Naturalistic Evaluation,” New Directions for Program Evaluation 1986, no. 30 (1986): 73–84.

[39] A. Sayani , L. Monteith , A. Shahil‐Feroz , et al., “Mobilizing the Power of Lived/Living Experiences to Improve Health Outcomes for All,” Health Expectations 28, no. 2 (2025): e70212. 40101202 10.1111/hex.70212 PMC11918782

[40] R. Ong ViforJ , R. Singh , E. Baker , R. Bentley , and J. Hewton , “Precarious Housing and Wellbeing: a Multi‐Dimensional Investigation. AHURI Final Report,” 2022.

[41] C. Stacy , T. R. Hodge , T. M. Komarek , et al., “Rent Control and the Supply of Affordable Housing,” Journal of Housing Economics 68 (2025): 102063.

[42] T. Hoření Samec , A. Decker , and L. Trlifajová , “Consumption Loans Consuming Homes: Intersections of Housing Precarity and Personal Overindebtedness,” International Journal of Housing Policy 24, no. 3 (2024): 501–520.

[43] World Health Organization , Ottawa Charter for Health Promotion (WHO, 1986), 10.1093/heapro/1.4.405.

[44] P. C. Williams , A. Binet , D. M. Alhasan , N. M. Riley , and C. L. Jackson , “Urban Planning for Health Equity Must Employ an Intersectionality Framework,” Journal of the American Planning Association 89, no. 2 (2023): 167–174.

[45] P. D. R. N. A. C. F. J. F. Kilanowski , “Breadth of the Socio‐Ecological Model,” Journal of Agromedicine 22, no. 4 (2017): 295–297. 28742433 10.1080/1059924X.2017.1358971

[46] J. Abood , M. Polonsky , K. Woodward , J. Green , Z. Tadjoeddin , and A. M. N. Renzaho , “Understanding Settlement Services Literacy and the Provision of Settlement Services for Humanitarian Migrants in Australia—A Service Provider Perspective,” Australian Journal of Social Issues 57, no. 3 (2022): 687–708.

[47] S. McShane , K. Block , and R. Bentley , “Harnessing Capital: The Role of Service Providers as Connectors, Supporters and Advocates in Refugee Housing and Settlement in Australia,” Journal of Immigrant and Refugee Studies 1‐16 (2025): 1–16.

[48] J. Broom , “An Australian Labor Party for the Future (Fund): The Politics of Financialisation in the Housing Australia Future Fund,” Australian Journal of Political Science 1‐20 (2026): 1–20.

[49] S. I. Daniels , H. Cheng , C. Gray , B. Kim , C. D. Stave , and A. M. Midboe , “A Scoping Review of Implementation of Health‐Focused Interventions in Vulnerable Populations,” Translational Behavioral Medicine 12, no. 9 (2022): 935–944. 36205470 10.1093/tbm/ibac025

[50] D. E. Velasquez , K. Mecklai , S. Plevyak , B. Eappen , K. A. Koh , and A. F. Martin , “Health System‐Based Housing Navigation for Patients Experiencing Homelessness: A New Care Coordination Framework,” Healthcare 10, no. 1 (2022): 100608. 34999493 10.1016/j.hjdsi.2021.100608

[51] T. Rong , E. Ristevski , and M. Carroll , “Exploring Community Engagement in Place‐Based Approaches in Areas of Poor Health and Disadvantage: A Scoping Review,” Health & Place 81 (2023): 103026. 37084705 10.1016/j.healthplace.2023.103026

[52] D. Rambaldini‐Gooding , L. Molloy , M. Barrington , et al., “Developing Professional Education to Support Cultural Humility in Maternal Health Care: Reflecting on a Co‐Design Project With Refugee and Migrant Women,” Midwifery 148 (2025): 104494. 40544671 10.1016/j.midw.2025.104494

[53] P. Harris , C. V. Robertson , B. Palmer , and A. Waters , “Working Together to Understand the Priority Needs of Multicultural Community Leaders in Queensland, Australia,” Journal of Intercultural Studies 46, no. 4 (2025): 681–699, 10.1080/07256868.2025.2531765.

[54] J. Abood , J. Green , M. J. Polonsky , K. Woodward , Z. Tadjoeddin , and A. M. N. Renzaho , “The Importance of Information Acquisition to Settlement Services Literacy for Humanitarian Migrants in Australia,” PLoS One 18, no. 1 (2023): e0280041. 36607991 10.1371/journal.pone.0280041 PMC9821785

