# Supplementary material for: Bridging Housing and Health Inequities: A Qualitative Study on Potential Solutions From the Perspectives of Recently Arrived Migrants and Refugees in Australia
Source: Health Promot J Austr. 2026 Jul 27;37(4):e70225. doi: 10.1002/hpja.70225 (PMC13403287; doi:10.1002/hpja.70225)
Supplement: Supplementary file 1 — Table S1: Consolidated criteria for reporting qualitative research (COREQ) 32‐item checklist. [file HPJA-37-0-s001.docx]

**Supplementary Materials:**

**Table S1.** Consolidated criteria for reporting qualitative research (COREQ) 32-item checklist

| **Item No. and Topic** | **Guide Questions/Description** | **Notes** |
| --- | --- | --- |
| **Domain 1: Research team and reﬂexivity** | | |
| *Personal characteristics* | | |
| 1. Interviewer/facilitator | Which author/s conducted the interview or focus group? | K.R. conducted all interviews |
| 2. Credentials | What were the researcher’s credentials? E.g. PhD, MD | K.R.: PhD Candidate |
| 3. Occupation | What was their occupation at the time of the study? | K.R.: PhD candidate |
| 4. Gender | Was the researcher male or female? | K.R.: Female |
| 5. Experience and  training | What experience or training did the researcher have? | The interviewer (K.R.) has prior experience in qualitative research |
| *Relationship with participants* | | |
| 6. Relationship  established | Was a relationship established prior to study commencement? | The interviewer (K.R.) had no relationship with the participants prior to the commencement of the study |
| 7. Participant knowledge  of the interviewer | What did the participants know about the researcher? e.g. personal goals, reasons for doing the research | The study aims and researchers involved were noted in the participant information sheet |
| 8. Interviewer  characteristics | What characteristics were reported about the inter viewer/facilitator? e.g. Bias, assumptions, reasons and interests in the research topic | The participant information sheet included the study aims and name and organisation of the researchers |
| **Domain 2: Study design** | | |
| *Theoretical framework* | | |
| 9. Methodological  orientation and Theory | What methodological orientation was stated to underpin the study? e.g. grounded theory, discourse analysis, ethnography, phenomenology, content analysis | Qualitative research design grounded in the constructivism paradigm, and thematic analysis involving an inductive approach |
| *Participant selection* | | |
| 10. Sampling | How were participants selected? e.g. purposive, convenience, consecutive, snowball | A combination of purposive and snowball sampling |
| 11. Method of approach | How were participants approached? e.g. face-to-face, telephone, mail, email | Interested participants were contacted via telephone and email |
| 12. Sample size | How many participants were in the study? | A total of 30 participants |
| 13. Non-participation | How many people refused to participate or dropped out? Reasons? | None of the 30 participants refused to participate or dropped out |
| *Setting* | | |
| 14. Setting of data  collection | Where was the data collected? e.g. home, clinic, workplace | Interviews were conducted via online videoconferencing (Zoom) |
| 15. Presence of non-  participants | Was anyone else present besides the participants and researchers? | Only the participant and interviewer were present |
| 16. Description of  sample | What are the important characteristics of the sample? e.g. demographic data, date | Participant characteristics are presented in Results |
| *Data collection* | | |
| 17. Interview guide | Were questions, prompts, guides provided by the authors? Was it pilot tested? | Semi-structured interview guide was used. It was pilot tested with initial participants. |
| 18. Repeat interviews | Were repeat interviews carried out? If yes, how many? | Repeat interviews were not carried out |
| 19. Audio/visual  recording | Did the research use audio or visual recording to collect the data? | All interviews were audio-recorded and transcribed verbatim |
| 20. Field notes | Were ﬁeld notes made during and/or after the interview or focus group? | Field notes were made after each interview |
| 21. Duration | What was the duration of the interviews or focus group? | Interviews were between 25 and 52 minutes (average 36 minutes) |
| 22. Data saturation | Was data saturation discussed? | Sample size was determined based on data saturation |
| 23. Transcripts returned | Were transcripts returned to participants for comment and/or correction? | Transcripts were returned to participants for comment and correction |
| **Domain 3: Analysis and ﬁndings** | | |
| *Data analysis* | | |
| 24. Number of data  coders | How many data coders coded the data? | One researcher (K.R.) coded the data |
| 25. Description of the  coding tree | Did authors provide a description of the coding tree? | Themes are presented in Results, and description of coding tree is not included |
| 26. Derivation of themes | Were themes identiﬁed in advance or derived from the data? | Themes were derived from the data |
| 27. Software | What software, if applicable, was used to manage the data? | NVivo 15 software was used to manage the data |
| 28. Participant checking | Did participants provide feedback on the ﬁndings? | Participant feedback on the findings was not collected |
| *Reporting* | | |
| 29. Quotations presented | Were participant quotations presented to illustrate the themes/ﬁndings? Was each quotation identiﬁed? e.g., participant number | Participant quotations are presented in Results. Quotations are identified through pseudonyms. |
| 30. Data and ﬁndings  consistent | Was there consistency between the data presented and the ﬁndings? | All findings were derived from the data and all themes are supported by quotes |
| 31. Clarity of major  themes | Were major themes clearly presented in the ﬁndings? | Major themes were derived from the data and are presented in Results |
| 32. Clarity of minor  themes | Is there a description of diverse cases or discussion of minor themes? | Description of diverse cases and discussion of minor themes are also presented in Results |

Developed from: Tong A, Sainsbury P, Craig J. Consolidated criteria for reporting qualitative research (COREQ): a 32-item checklist for interviews and focus groups. *International Journal for Quality in Health Care*. 2007. Volume 19, Number 6: pp. 349-357.
